# New Mode of Using Nitrate of Silver

**Published:** 1893-02

**Authors:** C. N. Peirce


### NEW MODE OF USING NITRATE OF SILVER.

TO THE EDITOR OF THE INTERNATIONAL DENTAL JOURNAL:

SIR,—I send you enclosed a piece of blotting-pad which has been saturated with a forty-per-cent. solution of silver nitrate. I have been trying lately numerous experiments to have a form of this caustic which could be applied to children's teeth without the direct application of the crystal, which is always attended with some danger, and liable to stain the fingers, napkins, and instruments.

This preparation seems to work very happily, and is of abundant strength *for all* purposes required in the mouth, whether for cauterizing the soft tissues or acting on the hard. It is well known that nitrate of silver is very soluble, dissolving in its own weight of water. This strong solution I tried first on some short fibre of cotton, but found, when dried, that the cotton was entirely destroyed. This strength—forty per cent.—is about as strong as it can be used without some destruction of the fabric. The pad, thus prepared, can be cut into small pieces, and be always ready for use, if it be kept dry.

C. N. Peirce.

[Remarks.—While we have not had an opportunity to test the sample sent, there can be no doubt of its value. It will unquestionably prove a great convenience in the application of nitrate of silver, and our readers, we are sure, will have reason to thank Professor Peirce for its timely introduction.—Ed.]

